# A pilot study of implication of machine learning for relapse prediction after allogeneic stem cell transplantation in adults with Ph-positive acute lymphoblastic leukemia

**DOI:** 10.1038/s41598-023-43950-w

**Published:** 2023-10-05

**Authors:** Kseniia S. Afanaseva, Evgeny A. Bakin, Anna G. Smirnova, Ildar M. Barkhatov, Tatiana L. Gindina, Ivan S. Moiseev, Sergey N. Bondarenko

**Affiliations:** Department of Bone Marrow Transplantation of Adults, RM Gorbacheva Research Institute, Pavlov University, Lev Tolstoy Str., 6/8, Saint-Petersburg, Russia 197022

**Keywords:** Acute lymphocytic leukaemia, Translational research

## Abstract

The posttransplant relapse in Ph-positive ALL increases the risk of death. There is an unmet need for instruments to predict the risk of relapse and plan prophylaxis. In this study, we analyzed posttransplant data by machine learning algorithms. Seventy-four Ph-positive ALL patients with a median age of 30 (range 18–55) years who previously underwent allo-HSCT, were retrospectively enrolled. Ninety-three percent of patients received prophylactic/preemptive TKIs after allo-HSCT. The values of the BCR::ABL1 level at serial assessments and over variables were collected in specified intervals after allo-HSCT. They were used to model relapse risk with several machine-learning approaches. GBM proved superior to the other algorithms and provided a maximal AUC score of 0.91. BCR::ABL1 level before and after allo-HSCT, prediction moment, and chronic GvHD had the highest value in the model. It was shown that after Day + 100, both error rates do not exceed 22%, while before D + 100, the model fails to make accurate predictions. As a result, we determined BCR::ABL1 levels at which the relapse risk remains low. Thus, the current BCR::ABL1 level less than 0.06% in patients with chronic GvHD predicts low risk of relapse. At the same time, patients without chronic GVHD after allo-HSCT should be classified as high risk with any level of BCR::ABL1. GBM model with posttransplant laboratory values of BCR::ABL1 provides a high prediction of relapse after allo-HSCT in the era of TKIs prophylaxis. Validation of this approach is warranted.

## Introduction

Philadelphia chromosome-positive (Ph-positive) acute lymphoblastic leukemia (ALL) is the most common subtype of ALL in adults, characterized by the abnormal formation of the Philadelphia chromosome, which leads to the development of the BCR::ABL1 gene with increased activity. The prognosis of these patients has changed dramatically since the successful incorporation of tyrosine kinase inhibitors (TKIs) into chemotherapy regimens^1^. At the same time, allogeneic hematopoietic stem cell transplantation (allo-HCT) is considered to improve the outcomes. It remains the standard consolidation strategy for achieving long-term survival according to current international recommendations, such as National Comprehensive Cancer Network (NCCN) (Version 1.2022) and European Bone Marrow Transplantation guidelines^2–4^. Unlike TKIs, allo-HSCT represents a multimodal immune therapy with 5-year overall survival (OS) rates ranging from 35 to 61%^5–8^. Despite the promising results achieved, relapse after allo-HSCT remains an unsolved problem. Historically, in case of posttransplant relapse, the prognosis of patients was extremely poor, and 1-year OS was about 10–20%. Salvage chemotherapy, donor lymphocyte infusions (DLIs), second allo-HSCT, TKIs with broader activity (second and third generations), monoclonal antibodies, and CAR-T therapy can be used as the potential options for relapse. Initial attempts are also being made to use asciminib in combination with TKIs to overcome the resistance of cell subclones^9–11^. The latest data of Acute Leukemia Working Party (ALWP) of the EBMT demonstrates that with the improvement of transplant-related factors, posttransplant salvage, and supportive care, the 2-year OS after relapse increased from 27.8% for patients relapsing between 2000 and 2004 to 54.8% for 2015 and 2019 (p = 0.001)^12^, which means that only half of the relapsed patients can be cured from the first posttransplant relapse. Thus, the strategy not to treat but to prevent posttransplant relapse seems more promising, but there is no standard approach. Nonetheless, most centers found a positive impact of posttransplant TKIs administration^13–15^. Moreover, there is no clear understanding of which clinical factors after allo-HSCT play a role in the development of relapse. For instance, BCR::ABL1 fluctuation after allo-HSCT in the context of posttransplant prophylactic TKIs administration is still a question. Thus, it seems optimistic and necessary to develop tools for calculating the risk of posttransplant relapse based on clinical and laboratory characteristics of the disease after allo-HSCT.

High sensitivity of a quantitative polymerase chain reaction (QT-PCR) makes it possible to change the treatment strategy before the development of relapse after allo-HSCT. However, there is a group of patients in whom molecular relapse and further fluctuation of BCR::ABL1 do not lead to a hematological relapse, which may be due to graft-versus-leukemia (GvL) effect. Standard statistical approaches have limited predictive power with longitudinal data. Several techniques may be applied for the classification of such data. One of these tools is the machine learning analytic approach that specializes in integrating of multiple risk factors into a predictive tool. Given the high variability of individual outcomes after allo-HCT and the importance of optimal patient management, we hypothesized that machine learning models may be precise, facilitate time-dependent relapse risk prediction after allo-HCT and support treatment decisions. This pilot study aims to apply modern machine learning approaches for building relapse predicting model in adult Ph-positive ALL patients after allo-HCT.

## Materials and methods

This single-center study was conducted with the retrospective cohort of 74 Ph-positive ALL patients who received allo-HSCT at RM Gorbacheva Research Institute, Pavlov University, between 2008 and 2021. Diagnosis of Ph-positive ALL was made according to 2016 World Health Organization criteria. Inclusion criteria were: 1. Age ≥ 18 years at allo-HSCT; 2. Patients undergoing first allo-HSCT and have follow-up to Day + 100 after allo-HSCT; 3. Engraftment and donor chimerism. 4. Complete clinical data and outcome data available; 5. Available data about BCR::ABL1 levels at different time intervals after allo-HSCT. The study was approved by the Pavlov University Ethical Committee and conducted ethically following the World Medical Association Declaration of Helsinki. All patients provided written informed consent to use their medical and personal data for research purposes.

The Consensus Conference criteria were used for acute graft-versus-host disease (GVHD) grading, and National Institutes of Health criteria were used for chronic GVHD grading^16,17^. Myeloablative conditioning (MAC) was performed with FB4 or treosulfan ≥ 42 g/m^2^. Reduced-intensity conditioning (RIC) included FB2-3 conditioning or melphalan doses of ≥ 140 mg/m^2^. The time intervals of data collection represent standard practices for bone marrow aspirations after allo-HCT.

Complete remission (CR) was defined as blast cell ratio < 5% at the ANC counts of > 1 × 10*9/L and platelet numbers of > 100 × 10*9/L. Molecular response (MR) or minimal residual disease (MRD) negativity was defined as undetectable BCR::ABL1 transcript level determined by Real-time qPCR. MRD was defined as detectable BCR::ABL1 p210 or p190 transcript level after remission induction or relapse treatment and was assessed for patients in CR only. BCR::ABL1 level is presented as a relative percent per xABL1 copies. A relapse was defined as the presence of > 5% of clonal blasts in bone marrow or any extramedullary site in the patients with previously documented CR.

Conventional cytogenetic analysis was used to evaluate of chromosome aberrations at diagnosis or during follow-up. Karyotypes were described according to an International System for Human Cytogenomic Nomenclature^18^. The interphase blast cells were evaluated using fluorescence in situ hybridization (FISH) probes designed for the detection of (9;22) translocation. Relative expression levels of BCR::ABL1 for p190 and p210 were measured using the standard qPCR approach and calculated as BCR::ABL1 level/ABL1 level*100%. The ABL1 gene was used for normalization of the results. The samples with at least > 10,000 copies of the reference ABL1 gene per reaction were considered valid.

OS was defined as the probability of survival, irrespective of the disease status, at any time point after allo-HSCT. RFS was defined as the probability of survival without relapse at any time point after allo-HSCT. Probabilities of OS and RFS were calculated using the Kaplan–Meier Method. The comparisons were made using the log-rank test. P-values are two-sided with a type 1 error rate fixed at 0.05. When RFS was calculated, death and relapse were defined as events. The RI was defined as cumulative incidence of disease relapse after allo-HSCT. NRM was defined as the cumulative probability of death without a relapse after allo-HSCT. Analysis of time-dependent variables, such as RI, NRM and GvHD incidence, were calculated using cumulative incidence estimates with a competing risk setting using the Fine and Grey test. A competing risk of RI was death without relapse and a competing risk of NRM was relapse, respectively. A competing risk of chronic GvHD was death without chronic GvHD. Patients alive at the end of the follow-up were censored at this date.

The secondary endpoint was to build a visual model for predicting relapse based on machine-learning approach. The methodology of the machine learning process is presented in the online Supplementary.

Statistical analyses were performed with EZR free statistical environment, version 2.15.2 (R Foundation for Statistical Computing, Vienna, Austria). Machine learning models were developed using R package caret v.6.0-90. (R Development Core Team, Vienna, Austria).

## Results

A total of 74 Ph-positive ALL patients with a median age of 30.5 (range 18–55) years were retrospectively included in the study. The median follow-up time was 26 (range 1.0–116.0) months for the patients who were still alive at the end of the study. Most of the allo-HSCT were performed in CR1 from matched unrelated donors (MUD) with RIC regimens and posttransplant cyclophosphamide GvHD prophylaxis. Most patients (n = 53, 82%) received posttransplant TKIs with the prophylactic aim. The median time from allo-HSCT to first relapse was 8 (range 3–63) months. Two (2.7%) patients had a relapse during the first 100 days after allo-HSCT. Relapse occurred in 25 (34%) of the patients: among them, isolated neuroleukemia developed in 2 (3%), other variants of extramedullary relapse in 3(4%), combined (CNS + bone marrow + other extramedullary) relapse in 3 (4%). Six (8%) patients experienced more than one relapse. By the end of the analysis, 53 patients (72%) were alive. It is essential to mention that relapse was the leading cause of death (n = 14, 67%) after allo-HSCT. The proportion of patients with mild chronic GvHD was 10 (14%), moderate chronic GvHD 11 (15%) and severe chronic GvHD 11 cases (15%). Other baseline characteristics of the patients and transplant procedure are presented in Table 1.

**Table 1:** Characteristics of the patients and allo-HSCT.

Characteristics	N (%)
Gender	
Male	47 (64)
Female	27 (36)
Protein type	
P190	60 (81)
P210	14 (19)
Status of the disease	
CR1	51 (69)
CR2	12 (16)
≥ CR3	5 (7)
Active disease	6 (8)
MRD status	
MRD-positive	35 (51)
MRD-negative	33 (49)
TKIs prior to allo-HSCT (first or second generation)	
Yes	70 (95)
No	4 (5)
Donor	
MUD	45 (61)
MRD	17 (23)
Haploidentical	12 (16)
Graft source	
Bone Marrow	19 (26)
PBSC	55 (74)
Conditioning regimen	
MAC	18 (24)
RIC	29 (40)
Non-MAC	27 (36)
GvHD prophylaxis	
PtCy-based	55 (75)
ATG-based	9 (12)
TCR αβ-depletion	4 (5)
Other	6 (8)
TKIs after allo-HSCT	
Imatinib	31 (42)
Dasatinib	28 (37)
Bosutinib	2 (3)
Nilotinib	1 (2)
Switch to another TKIs	3 (4)
No TKIs	9 (12)
Purpose of TKIs administration	
Prophylactic	53 (82)
Preemptive	7 (11)
Relapse treatment	5 (7)
TKIs in combination with	
Donor lymphocytes infusion (DLI)	9 (14)
Radiation therapy	1 (2)
Endolumbar cytostatic injections	3 (4)
Surgical treatment (orchiectomy)	2 (3)
TKIs as a sole agent	50 (77)
Type of relapse after allo-HSCT	
Isolated bone marrow	17 (23)
With extramedullary organs (CNS or testicles)	8 (11)
Chronic «graft-versus-host» disease	
Yes (any grade)	32 (43)
No	42 (57)

*MUD* matched unrelated donor, *MRD* matched related donor, *PBSC* peripheral blood stem cells, *PtCy* posttransplant cyclophosphamide, *ATG* anti-thymocyte globulin, *TCR αβ-depletion* T cell receptor alpha/beta-depletion.

The cumulative incidence of relapse and NRM at five years were 38.8% (95% CI 26.3–51.3) and 10.0% (95% CI 2.2–17.8), respectively. The Kaplan–Meier OS estimate at five years was 67.7% (95% CI 55.4–80.0), and the estimate of RFS at five years was 55.0% (95% CI 42.7–76.3). The cumulative incidence of NIH-defined chronic GVHD was 46.4% (95% CI 33.9–58.0%), with a median onset time of 199 (range 101–1041) days. The disease status was a significant risk factor for NRM and amounted to 7.0% (95% CI 3.0–10.9%) in CR1 versus 19.0% (95% CI 2.8–35.1%) in other statuses (p = 0.05). Primary analysis of factors influencing RI and RFS was performed in univariate analysis for patients who were transplanted in CR. Among factors that decreased the probability of RFS are disease status beyond CR1, MRD-positive status prior to allo-HSCT, and lack of TKIs prophylaxis after allo-HSCT (p < 005).

To create a tool for relapse prediction, we included in the model time intervals between allo-HSCT and prediction moment, BCR::ABL1 expression level at prediction moment, therapy after allo-HSCT, the highest BCR::ABL1 expression level before prediction moment, and the chronic GvHD status before prediction. For the analysis, all TKIs were divided into two groups TKIs1: imatinib, TKIs2—other TKIs, regardless of generation. All time intervals for which BCR::ABL1 level values had been collected were grouped into three significant time intervals: before Day + 100, Day + 100–250 and after Day + 250 solely for data presentation.

**Figure 1:**
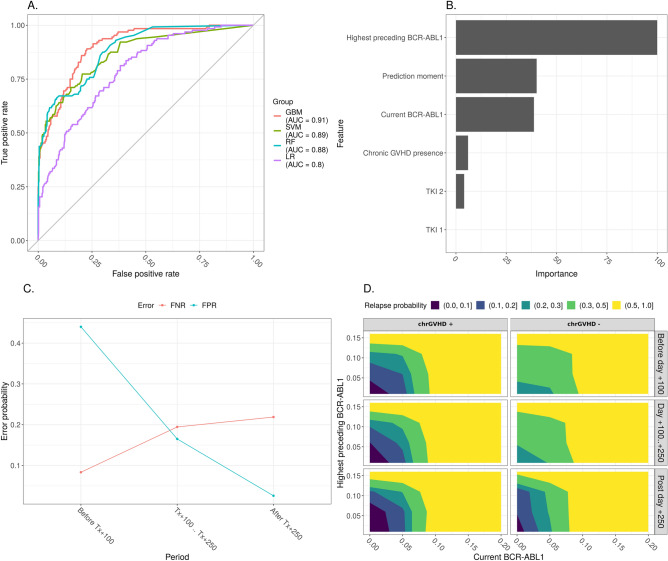
(**A**) Various machine-learning algorithms used. (**B**) Variable importance plot for the GBM algorithm. (**C**) Error plot for the GBM algorithm. (**D**) Guiding map (visualization of relationship between relapse probability and factors values). *Notes*: (**B**) Variable importance plot provides a list of the most significant variables in descending order: the top variables contribute more to the model than the bottom ones and have high predictive power in classifying default and non-default customers. (**D**) Each color corresponds to a certain risk of relapse: black area corresponds to the risk of relapse from 0 to 10%, aquamarine area—from 10 to 20%, jade areas—from 20 to 30%, green area—from 30 to 50%, yellow area—from 50 to 100%.

To build a classification model, four classification algorithms were applied: Logistic regression, Random Forest, Support vectors machine, and Gradient Boosting Method (GBM). The resulting ROC curve for the most effective classification models is shown in Fig. 1A. Classification accuracy is a metric used to evaluate the performance of a model based on the predicted class labels and is a good starting point for many classification tasks. AUC (areas under curves) is another effective indicator for assessing the prediction model’s discrimination. GBM provided a maximal AUC score (0.91) in our group. For this, a decision-making threshold may be adjusted to obtain a specificity of about 0.75 and a sensitivity of about 0.91. This means that using GBM, we have an opportunity to predict a relapse with a sensitivity of 0.91, i.e., predicting a relapse in those who will experience it (true positive rate) and 25%—the fraction of patients in whom it actually will not occur (1—false positive rate). The median time from prediction moment to relapse was 108 days (range 15–491).

In our model highest BCR::ABL1 level, the time of prediction moment, chronic GvHD, and current BCR::ABL1 level have the most robust importance (Fig. 1B). At the same time, TKIs prophylaxis turned out to be a less significant factor, which may be a consequence of the fact that the majority of the patients received TKIs, and the group was homogeneous in terms of this variable (Fig. 1B; Supplementary material, Fig. S1).

When analyzing the model accuracy, false-negative and false-positive rate errors were estimated for the three ranges of prediction moments as mentioned above (Fig. 1C). It was determined that after Day + 100, both error rates do not exceed 22%. In contrast, before Day + 100, the model fails to make an accurate prediction based on the independent variables used.

The following guiding maps were calculated for practical decision-making process using this model (Fig. 1D). The color of the area corresponds to the probability of relapse. The scale «current BCR::ABL1» and «highest preceding BCR::ABL1», which also includes MRD levels before allo-HSCT, show the relative values ​​of the BCR::ABL1 transcript after allo-HSCT. According to the map, depending on the presence or absence of chronic GVHD and the definite levels ​​of the BCR::ABL1, the patient can be assigned to a group of high (the relapse risk is over 20%) or low (the relapse risk is less 20%) risk of relapse. Thus, since day + 100 after allo-HSCT the patients with chronic GvHD and current BCR::ABL1 level equal to or higher than 0.06% can be classified as high risk of relapse. If such a patient has current BCR::ABL1 level less than 0.06% and simultaneously he had had BCR::ABL1 level equal to or higher than 0.11% previously at any time after allo-HSCT, he is also at a high risk of relapse. At the same time, if the patient had no chronic GVHD after allo-HSCT till the prediction moment, he should be classified to a high risk group at any BCR::ABL1 level. According to the data, in the context of either prophylaxis or preemptive TKIs, the group of patients which is not required additional treatment or intervention after allo-HSCT in case of BCR::ABL1 appearance can be defined.

## Discussion and conclusion

Despite improvement in outcomes in recent years, high RI rates after allo-HSCT remain the leading cause of transplant failure and limit the curative potential of this therapy^19^. For the Ph-positive ALL group, the impact of various factors that have a negative influence on the prognosis of the disease, such as additional chromosomal abnormalities, a mutational profile outside the BCR::ABL1 gene and several others were previously described^20–22^. However, there is limited data on the effect of these factors in the context of chronic GVHD and posttransplant TKIs prophylaxis. Also, pre-transplant factors only usually have low predictive power.

A machine learning is a data-driven analytic approach that allows several factors to be integrated into a predictive model. It has been successfully used in several cancers, including adult leukemia, and predicts relapse and survival in different settings^23–26^. We performed the retrospective study based on machine learning technologies of developing an empirical model using existing clinical and specific molecular time-dependent variables associated with Ph-positive ALL. The model may help physicians to react earlier to critical events and to predict relapse after allo-HSCT in the context of TKIs maintenance.

Most of the models in the field of allo-HSCT that use pretransplant data, even with neural network training, have low predictive ability (typically AUC 0.6–0.75), which is the main obstacle for their clinical application^27–30^. At the same time, certain studies focus not on outcomes but on the prediction of donors’ availability with an AUC of 0.826^31^. On the other hand, the problem of relapses was addressed by several studies with machine learning methods in pediatric ALL^32,33^. In some of them, the AUC reaches acceptable for practical application levels: in the study by Pan et al., the model demonstrated a high AUC level of 0.904^34^. Interestingly, the number of studies based on posttransplant variables is even more limited: in one of them by Eisenberg et al., machine models provided well-calibrated, time-dependent risk predictions and achieved appropriate levels of AUC of 0.92 and 0.83 for prediction of mortality and CMV reactivation, respectively, in a 21-day time window after allo-HSCT^35^. However, to our knowledge, there are no reliable machine-learning approaches to predict relapse in adult Ph-positive ALL patients after allo-HSCT. We built the prediction model and created an illustrated map for both posttransplant bone marrow and combined relapses with the extramedullary involvement with high sensitivity and reasonable specificity based on the relatively limited group of patients with TKIs maintenance, which accurately predicts relapses in the interval of Day + 100–250 when most of the relapses occur.

It should be noted that a precise clinical strategy regarding the posttransplant TKIs prophylaxis has still not been described. Most of the surveys confirm the positive impact of posttransplant TKIs prophylaxis despite controversial data obtained in other studies^14,15,36–42^. We also found a beneficial effect of prophylactic use of TKIs after allo-HSCT in our test cohort of patients. Moreover, it was described previously that TKIs prophylaxis improves long-term RFS and alleviates the negative impact of MRD on the outcomes in Ph-positive ALL adult patients^43^. As we demonstrated, the therapy (TKIs1/TKIs2) is the least important variable in this model, which may be because more significant proportion of patients (82%) in the study received prophylactic TKIs after allo-HSCT.

Situations of high relapse risk require additional medical intervention to prevent relapse: there are several treatment options in case of posttransplant relapse. It is known that with salvage conventional chemotherapy the CR rate is low (30–40%). Also poor long-term outcome can be expected^44–46^. Nowadays, the best option of relapse treatment consists of the change of TKIs according to the mutation profile or use of immunotherapy. If no mutation analysis could be performed, using third generation TKIs is the best option because T351I positive relapses accounted for 71% of all relapses^47^. Moreover, immunotherapy with blinatumomab has proven to be effective as a single drug in r/r Ph-positive ALL in the Phase II ALCANTARA study. It showed promising results with a median OS of 7.1 months and RFS of 6.7 months, 16 out of the 45 (36%) patients achieved CR, 14 (86%) patients achieved complete molecular remission (CMR)^48^. In the Phase III randomized INO-VATE study, inotuzumab ozogamicin showed favorable rates of CR compared to standard chemotherapy (78.6% vs. 44.4%, p = 0.08)^47^. There are also several studies revealing the efficacy of the combination of blinatumomab and a second or third generation TKIs, which demonstrate reasonable clinical outcomes. When using the combination of blinatumomab and ponatinib, CR and CMR rates were 96.2% and 88.5%, respectively. The 2-year OS and RFS rates were 41.4%, and 31.8%^49^. Another potential treatment option is CAR-T therapy. Thus, in the Phase II ELIANA trial, which enrolled 75 children and adolescents with r/r B-ALL, the CR rate was 81%, and 1-year OS and DFS were 76% and 50%, respectively. Several patients with Ph-positive ALL were enrolled in these trials, but no subgroup analysis is available^50^. By now, further investigations are needed for Ph-positive ALL patients in the area of CAR-T cells. Taking into account the above, it should be noted that immunotherapy may represent an encouraging prospect not only in treatment but also in the prevention of posttransplant relapse in the setting of TKIs maintenance. Blinatumomab is currently registered for the treatment of MRD-positive status. Furthermore, there are already ongoing studies on the effectiveness of blinatumomab after allo-HSCT as maintenance^51^.

Fluctuations of BCR::ABL1 in Ph-positive malignancies may occur after transplantation. Though the criteria of molecular relapse is defined as detectable BCR::ABL1 transcript level by Real-time qPCR confirmed by at least two consecutive tests after previous molecular response. No recommendations exist on the critical BCR::ABL1 that should be considered as molecular relapse and require intervention. In the setting of prophylaxis/preemptive TKIs we observed that low-level fluctuations do not necessarily lead to a relapse and do not require additional treatment. Moreover, chronic GVHD allows more significant fluctuations, which confirms GvL for this type of blood cancer^52,53^.

At the same time, there are several significant limitations: despite the fact we get rather high AUC in the model, it is very likely that the sensitivity of the model will rise additionally, if we increase the patient population. In performed analysis the number of included patients is limited by the study inclusion criteria and prevalence of the disease in population, which makes it difficult to form a validation cohort for cut-off levels of BCR::ABL1. Due to these reasons only cross-validation has been performed at the current step of research. A multicenter validation is required to confirm the results of this study, but it seems difficult to organize because there are no p190 interlaboratory validation standards as for p210^54^. Thus, even cut-off values obtained in a centralized laboratory will not apply to local standards. If a larger group of patients is recruited, a sort of medical calculator can be created for the routine practice after validation. Second limitation is that the independent variables applied do not allow the model to predict isolated extramedullary relapses (which accounts for 7% in our cohort). At the same time, according to the literature data, the incidence of posttransplant isolated extramedullary relapses is relatively high. It varies from 5 to 15% in ALL patients^46,55–58^, which poses a significant challenge for physicians because of limited treatment options and low response rates in this refractory subgroup. For these patients, it is necessary to apply other variables based on the data of lumbar punctures and instrumental methods of research (magnetic resonance imaging, ultrasound, PET-CT and others).

In conclusion, this study identified that machine learning can be used for prediction of the posttransplant relapses in Ph-positive ALL. The study highlights that with TKIs prophylaxis, low-levels of BCR::ABL1 transcript fluctuations do not lead to relapse in some cases: it seems that patients who have chronic GvHD, do not require additional posttransplant therapy when low-levels of BCR::ABL1 appears. On the contrary, even low levels of BCR::ABL1 require augmented therapy if there is no chronic GvHD. This is a pilot study that was carried out as a proof of concept for the subsequent initiation of similar research in multicenter setting. It seems that the validation of the model is possible only in cooperative group research.

### Supplementary Information

Supplementary Information.

## Data Availability

All data generated or analyzed during this study are included in this article. Further enquiries can be directed to the corresponding author.
