# Supplementary material for: A pilot study of implication of machine learning for relapse prediction after allogeneic stem cell transplantation in adults with Ph-positive acute lymphoblastic leukemia
Source: Sci Rep. 2023 Oct 5;13:16790. doi: 10.1038/s41598-023-43950-w (PMC10556079; doi:10.1038/s41598-023-43950-w)
Supplement: Supplementary file 1 — Supplementary Information. [file 41598_2023_43950_MOESM1_ESM.pdf]

Supplement A. A machine learning model for posttransplant relapse prediction

The values of the BCR::ABL1 level at serial assessments of bone marrow samples of each patient were grouped into the following time intervals after allo-HSCT: Day+30-60; 61-90; 91-130; 131-180; 181-250; 251-400; 401-550; 551-600; 601-700; 701-1000; 1001-1500; 1501-2000; after 2001. In addition, an applied therapy (TKIs1 and TKIs2) was indicated for each time interval. The analysis was performed in three steps: data preparation, model building and model quality assessment.

Data preparation

Data preparation has the following peculiarities:

1. Each patient *i* data was split into M_i independent observations each of which represented a particular time point. Thus, the total number of $\sum_i M_i$ observations presented in the final data set.
2. Observations were characterized with the following independent variables: time after allo-HSCT, current level of BCR::ABL1, highest level of BCR::ABL1 in preceding period, chronic GVHD status and applied therapy. Missing values for continuous variables were imputed with their medians.
3. The dependent variable was derived as an existence of relapse in the time period following after current time point.

Model building

Model building was performed in *R* package *caret v.6.0-90*. The following machine learning algorithms were evaluated:

1. logistic regression (LR)
2. random forest (RF)
3. support vectors machine (SVM)
4. gradient boosting machines (GBM)

Model quality assessment

Each algorithm was cross-validated using k-fold algorithm (k = 5). The resulting ROC-curves are given in Figure 1A. According to the analysis, GBM provides the best accuracy relatively to other approaches (AUC=0.91). For the GBM the variable importance plot was build (Fig. 1B). Because the impact of therapy (TKIs1 and TKIs2) demonstrated the least importance in the model, we made an attempt to remove the fact of therapy from the data and train the model again. According to Figure S1, AUC lost its noticeable for GBM, so for the following analysis the previous full model was used.

Figure S1. ROC-curves for the models with excluded TKIs1/TKIs2

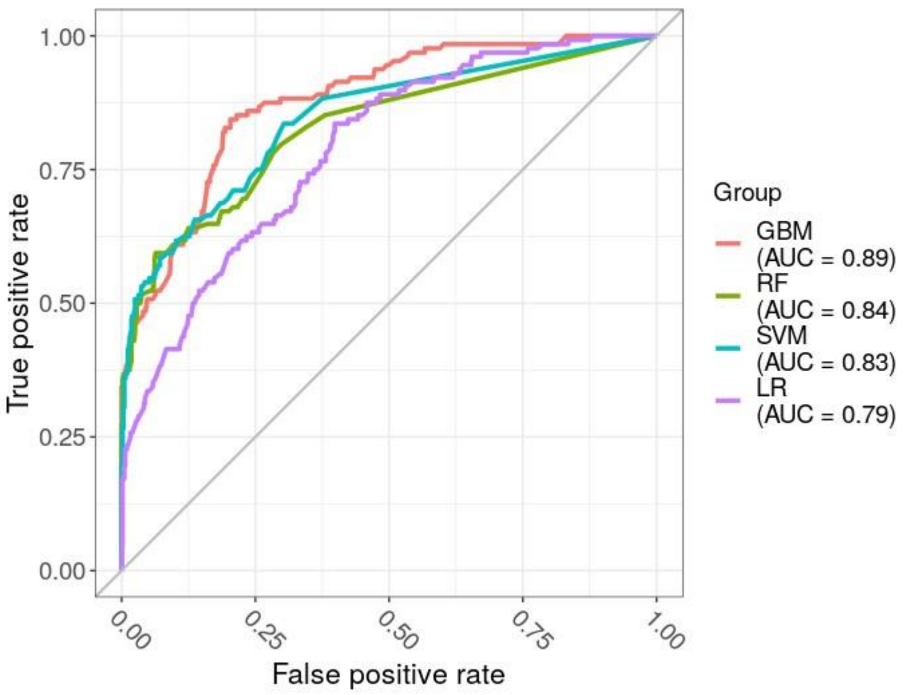

At the next step of analysis dependence of false positive/negative rate versus time for the chosen GBM model was evaluated (see Fig. 1C). For the analysis time intervals were grouped according to a predefined ranges 0-100, 100-250, 250+ days after allo-HSCT. The relationship between relapse probability and factors values is given in Fig. 1D, where the color indicates predicted relapse probability according to the chosen model.
